# Interleukin-10 Promoter Gene Polymorphisms and Susceptibility to Asthma: A Meta-Analysis

**DOI:** 10.1371/journal.pone.0053758

**Published:** 2013-01-15

**Authors:** Myung-Han Hyun, Chung-Ho Lee, Min-Hyung Kang, Bong-Kyung Park, Young Ho Lee

**Affiliations:** 1 Korea University College of Medicine, Seoul, Korea; 2 Division of Rheumatology, Department of Internal Medicine, Korea University Anam Hospital, Korea University College of Medicine, Seoul, Korea; University of Texas Health Science Center at San Antonio, United States of America

## Abstract

**Objective:**

The aim of this study was to explore whether the interleukin (IL)-10 polymorphisms and their haplotypes contribute to asthma susceptibility.

**Methods:**

MEDLINE, EMBASE and the COCHRANE library databases were utilized to identify available articles. A meta-analysis was conducted on IL-10 -1082 G/A, -819 C/T, -592 C/A polymorphisms, and their haplotypes and asthma.

**Results:**

Eleven studies involving 2,215 asthma patients and 2,170 controls were considered in the meta-analysis. The meta-analysis revealed no association between asthma and the IL-10 -1082 G allele [Odds ratio (OR) = 0.87, 95% Confidence interval (CI) = 0.68–1.12, p = 0.28]. However, meta-analysis of the five studies in Hardy-Weinburg equilibrium produced the relationship between the IL-10 -1082 G allele and asthma (OR = 0.71, 95% CI = 0.60–0.83, p<0.0001). Stratification by ethnicity indicated an association between the IL-10 -1082 G allele and asthma in East Asians (OR = 0.74, 95% CI = 0.57–0.96, p = 0.02), but not in West Asians. Furthermore, stratification by age indicated an association between the IL-10 -1082 G allele and asthma in adults and mixed groups (OR = 0.77, 95% CI = 0.62–0.96, p = 0.02; OR = 0.67, 95% CI = 0.49–0.92, p = 0.01). No association was found between asthma and IL-10 -819 C/T and IL-10 -592 C/A polymorphisms and their haplotypes.

**Conclusion:**

The IL-10 -1082 G/A polymorphism confers susceptibility to asthma in East Asians and in adults. However, the IL-10 -819 C/T, -592 C/A polymorphisms and their haplotypes are not associated with asthma.

## Introduction

Asthma is a chronic respiratory disease that is characterized by variable airway obstructions caused by bronchial hyperreactivity [1]. The development of asthma is determined by the interaction between host genetic susceptibility and environment [2]. Although the etiology of asthma remains unknown, an imbalance between T-helper type 1 (Th1)/Th2 paradigm has a dominating genetic component. Regulatory T (Treg) cells play a key role in balancing Th1/Th2 level [3], [4]. Treg cells contribute to immune regulating and anti-inflammatory responses due to down-regulation of antigen and macrophage activation through various proteins, including interleukin (IL)-10 [5], [6].

The IL-10 gene maps to 1q31-32. Promoter region polymorphisms appear to be correlated with variations in transcription. Three of several polymorphic sites within the promoter region of IL-10 have been described in some detail. These are -1082 G to A (rs1800896), -592 C to A (rs1800872), and -819 C to T (rs1800871) [7], [8]. The -1082 G/A and -592 C/A polymorphisms lie within a putative negative regulatory region that is a binding site of Ets transcription factors and STAT-3, respectively [9], [10]. On the other hand, the -819 C/T polymorphism presents a dimorphic polymorphism and lies within a putative positive regulatory region [7]. These polymorphisms exhibit strong linkage disequilibrium and appear in three major haplotypes (GCC, ACC and ATA) [11]. The GCC haplotype has been associated with exuberant production of IL-10, while the ATA haplotypes are associated with low levels of IL-10 production in cultured peripheral blood mononuclear cells [12], [13].

Accordingly, IL-10 production could be affected by polymorphisms that alter the binding site of transcription factors. Therefore, identifying plausible candidate genes to be excluded and causative genes to be further investigated with reliability is required. Various studies from different populations have been carried out to assess the association between IL-10 polymorphisms and asthma, either at the genotypic or the haplotypic level. Conflicting results were reported [14]–[24]. Most studies used a small sample size and lacked statistical power to obtain reliable conclusions. By using all the available published data to increase statistical power, resolve inconsistencies, and reduce the likelihood that random errors are responsible for false-positive or false-negative associations, we turned to meta-analysis [25]–[27].

In the current study, we present the results of a comprehensive search of literature and meta-analysis to explore whether the IL-10 -1082 G/A, -819 C/T, -592 C/A polymorphisms, and their haplotypes contribute to asthma susceptibility.

## Methods

### Identification of Eligible Studies and Data Extraction

We conducted an exhaustive search for studies that examined the association of IL-10 polymorphisms with asthma. We utilized the MEDLINE, EMBASE and the Cochrane Central Register of Controlled Trials (CENTRAL) to identify available articles published up to August 2012 in which IL-10 polymorphisms were studied in asthma patients and controls. The Medical Subject Heading (MeSH) terms and/or text words that were entered were ‘asthma’ or ‘bronchial spasm’ or ‘respiratory hypersensitivity’ or ‘wheezing’, and in combination with ‘polymorphism’ or ‘variant’ or ‘genotype’ or ‘allele’ or ‘mutation’, and in combination with ‘interleukin 10’ or ‘interleukin-10’ or ‘IL-10’or ‘IL10’ (Supplement S1). Furthermore, all references mentioned in the identified original articles were reviewed by hand-searching in order to investigate additional literature that was not indexed. Genetic association studies that observed the distribution of IL-10 genotypes in asthma patients and controls were eligible for inclusion. Studies were included in this meta-analysis if (1) they were case-control studies, (2) they contained original data and (3) they provided enough data to calculate odds ratios (ORs). There are no restrictions were placed on language, race, ethnicity or geographic area. We excluded the following: (1) studies containing overlapping data; (2) studies in which the number of null and wild genotypes or alleles could not be ascertained and (3) studies in which family members had been studied because their analysis was based on linkage considerations. The PRISMA Checklist and the flow chart for the studies are shown as Supplement S2 and Supplement S3 in the supporting information.

Data were extracted from original studies by two independent reviewers (M.H.H. and C.H.L.) regarding the methods and results of meta-analysis. Discrepancy between the reviewers was resolved by consensus or a third reviewer (Y.H.L). From each study, we extracted following information: first author, year of publication, racial descent and age of the study population, the number of cases and controls, definition of asthma patients, and genotype and allele frequency information for IL-10 polymorphisms.

### Quality Score Assessment

The quality of studies was also independently assessed by the two reviewers (M.H.H. and C.H.L.) who used quality assessment scores of molecular association studies of asthma recommended by Thakkinstian et al (Supplement S4) [28]. These scores were based on both traditional epidemiologic considerations and genetic issues [29]. Total scores ranged from 0 (worst) to 15 (best). Any disagreement was adjudicated by a third author (Y.H.L.). Low quality studies, quality score ≤4, were excluded.

### Evaluation of Publication Bias

Funnel plots are commonly used to detect publication bias. However, this method requires a range of studies with varying sizes and subjective judgments. Due to these limitations, we evaluated publication bias using Egger’s linear regression test [30], which measures funnel plot asymmetry using a natural logarithm scale of odds ratios (ORs).

### Statistical Analysis

Allele frequencies at the IL-10 polymorphisms were determined by the allele counting method. The Chi-square test was used to determine if observed frequencies of genotypes in controls conformed to Hardy-Weinberg (H-W) expectations.

Meta-analysis was executed using (1) allelic contrast and (2) recessive, (3) dominant and (4) homozygote contrasts. Point estimates of risks, ORs, and 95% confidence intervals (CI) were estimated for each study. Cochran’s Q-statistic was also used to assess within- and between study variations and heterogeneity was quantified using *I^2^*, which ranges from 0 to 100% and represents the proportion of between-study variability attributable to heterogeneity rather than chance [31]. *I^2^* values of 25, 50, and 75% were nominally considered low, moderate, and high estimates. The fixed effects model assumes that a genetic factor has a similar effect on asthma susceptibility across all studies are caused by chance alone [32]. Alternatively, the random effects model assumes that different studies shows substantial diversity and assesses both within-study sampling errors and between-study variances [33]. If the result of the heterogeneity was test was p>0.10, ORs were pooled according to the fixed effect model. Otherwise random effect model was used. Statistical manipulations were performed using a Comprehensive Meta-analysis computer program Review Manager 5.1 (Cochrane Collaboration, London, UK).

## Results

### Studies Included in the Meta-analysis

**Figure 1:**
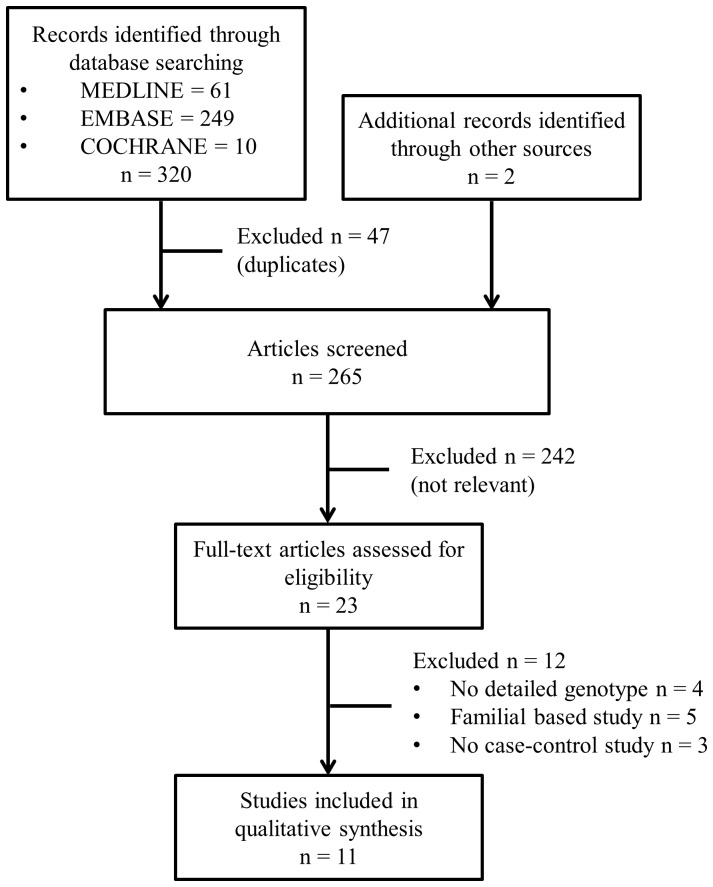
Study flow chart.

The flow diagram in Figure 1 outlines of selection process. Briefly, a total 263 articles were identified after eliminating 47 duplications. Two designated reviewers independently excluded definite irrelevant articles. Twenty three articles were enrolled for peer review. After review of the full-text articles, 12 publications were excluded from the meta-analysis for the following reasons: four did not report detailed genotype frequencies, five had family-based study designs, and three did not report case–control studies. Consequently, a total of 11 eligible publications were included in this meta-analysis with a total sample size of 2215 asthma subjects and 2170 control subjects (Table 1, Figure 1). Three were performed in European populations, three in East Asian populations, four in West Asian populations, and one in an Indian population. Four involved children alone, three recruited adults, and four included both children and adults. An ethnicity-specific meta-analysis was conducted on European, East Asian, and West Asian populations. Age stratification was performed on adult, children, and mixed subjects. Details of the IL-10 polymorphisms studies are summarized in Table 1. Quality assessment results are described in Supplement S5.

**Table 1:** Characteristics of the individual studies included in the systemic review and meta-analysis.

Study [Ref]	Population	Numbers		Age	Asthma definition	Studied polymorphism	Findings†	Quality score‡
		Asthma	Control					
Hakimizadeh et al. 2012 [14]	Iran (WA)	100	100	Mixed	ATS criteria	−592 C/A	−592 C allele (OR = 3.88, P = 0.001)	11
Hussein et al. 2011 [15]	Egypt (WA)	220	110	Children	ATS criteria	−1082 G/A	−1082 A allele (OR = 1.7, P = 0.01 in atopic asthma, OR = 1.6, P = 0.01 in non-atopic asthma)	8
Kim et al. 2011 [16]	Korea (EA)	333	248	Children	ATS criteria	−1082 G/A, −819 C/T, −592 C/A, Haplotype*	−1082 G/A allele (P = NS), −819 C/T (P = NS), −592 C/A (P = NS), Haplotype (P = NS)	9
Trajkov et al. 2009 [17]	Macedonia (EU)	74	301	Adult	NIH criteria	−1082 G/A, −819 C/T, −592 C/A, Haplotype*	−1082 GG vs. GA+AA(OR = 2.896, P = 0.007), −819 C/T (P = NS), −592 C/A (P = NS), Haplotype (P = NS)	7^§^
Movahedi et al. 2008 [18]	Iran (WA)	60	140	Children	NAEP guideline	−1082 G/A, −819 C/T, −592 C/A, Haplotype*	−1082 G allele (OR = 1.83, P = 0.008), −819 T allele (OR = 2.30, P = 0.001), −592 A allele (OR = 2.37, P = 0.001), ATA haplotype (OR = 2.33, P = 0.001)	7^§^
Zedan et al. 2008 [19]	Egypt (WA)	69	98	Children	Symptoms, GINA guideline	−1082 G/A	−1082 G/A allele (NS), GG vs. GA+AA (OR = 7, 95% CI = 2.4−20, P<0.001)	9^§^
Chatterjee et al. 2005 [20]	India (I)	273	307	Adult	NAEP guideline	−1082 G/A, −819 C/T, −592 C/A, Haplotype*	−1082 A allele (OR = 1.267, P = 0.03), − 819 C/T (P = NS), −592 A allele (OR = 1.318, P = 0.04), ATA haplotype (OR = 1.385, P = 0.0085)	11
Park et al. 2004 [21]	Korea (EA)	532	170	Mixed	ATS criteria	−1082 G/A, −592 C/A, Haplotype*	−1082 G/A (P = NS), −592 C/A (P = NS), ACC Haplotype vs. others (P = NS)	12
Karjalainen et al. 2003 [22]	Finland (EU)	242	395	Adult	Doctor’s diagnosis	Haplotype*	Haplotype (P = NS)	9
Hang et al. 2003 [23]	Taiwan (EA)	117	47	Mixed	Symptoms, PFT	Promoter -592 C/A	−592 AC vs. CC (OR = 1.833, 95% CI = 1.675–13.940), AA vs. CC (OR = 3.599, P = 0.013)	7
Lim et al. 1998 [24]	UK (EU)	195	241	Mixed	Symptoms, Asthma severity	Haplotype*	ATA haplotype vs. others (OR = 1.5, P = 0.01 in severe asthma, P = NS in mild asthma)	6

*Ref* references, *Vs.* versus, *NS* not significant, *WA* West Asian, *EA* East Asian, *EU* European, *OR* odds ratio, *CI* confidential interval, *PFT* pulmonary function test, *ATS* american thoracic society, *NAEP* national asthma education and prevention program, *GINA* global initiative for asthma, *NIH* national institute of health.

† Odds ratio indicated asthma versus control group.

‡ Quality assessment scoring system according to Thakkinstian et al. [28], ^§^Hardy-Weinberg equilibrium in deviation in control groups.

* Haplotype of IL-10 -1082 G/A, -819 C/T and -592 C/A.

### IL-10 -1082 G/A Polymorphism and Asthma

**Figure 2:**
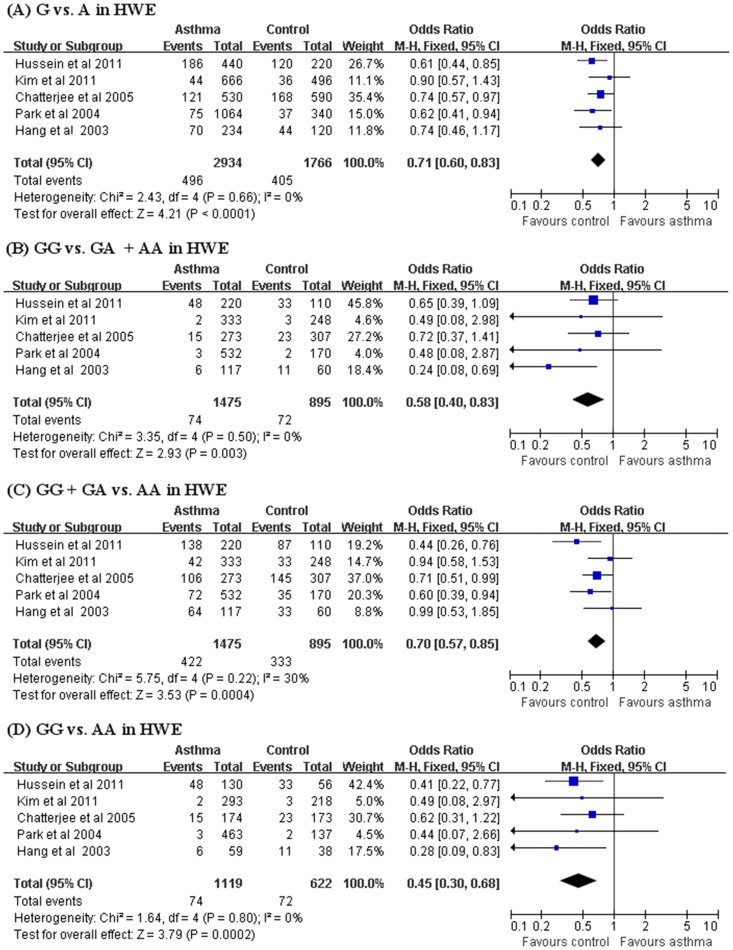
ORs and 95% CIs of individual studies and pooled data for the association between IL-10 -1082 G/A polymorphism and asthma for studies in Hardy-Weinberg equilibrium. (A) G vs. A allele (B) GG vs. GA+AA (recessive) (C) GG+GA vs. AA (dominant model) (D) GG vs. AA (Homozygote contrast).

A summary of the meta-analysis findings concerning associations between IL-10 -1082 G/A polymorphism and asthma is provided in Table 2. Meta-analysis of the eight case-control studies (1678 asthma and 1434 control subjects) revealed no association between asthma and the IL-10 -1082 G allele (OR = 0.87, 95% CI = 0.68–1.12, p = 0.28) (Table 2). In addition, analysis using the recessive, dominant model, and homozygote contrast showed the same pattern for the IL-10 -1082 G allele. However, the meta-analysis of the five studies in H-W equilibrium produced results for the relation between the IL-10 -1082 G allele and asthma (OR = 0.71, 95% CI = 0.60–0.83, p<0.0001) (Table 2, Figure 2). Analysis using the recessive, dominant model, and homozygote contrast showed the same pattern for the IL-10 -1082 G allele, showing an association between IL-10 -1082 G/A polymorphism and asthma (Table 2, Figure 2). Stratification by ethnicity indicated an association between the IL-10 -1082 G allele and asthma in East Asian (OR = 0.74, 95% CI = 0.57–0.96, p = 0.02) (Table 2). Analysis using the recessive model and homozygote contrast showed the same pattern for the IL-10 -1082 G allele (Table 2). However there was no association between IL-10 -1082 G/A polymorphism and asthma in West Asian (Table 2). Furthermore, stratification by age indicated an association between the IL-10 -1082 G allele and asthma in adults and mixed groups (OR = 0.77, 95% CI = 0.62–0.96, p = 0.02; OR = 0.67, 95% CI = 0.49–0.92, p = 0.01), but not in Child (OR = 1.07, 95% CI = 0.64–1.76, p = 0.80) (Table 2).

**Table 2:** Meta-analysis of association between the IL-10 -1082 G/A polymorphism and asthma.

Population	No		−1082 G/A G vs. A			GG vs. GA+AA (Recessive)			GG+GA vs. AA (Dominant)			GG vs. AA (Additive)		
			OR (95% CI)	P_Eff_	P_Het_ †	OR (95% CI)	P_Eff_	P_Het_ †	OR (95% CI)	P_Eff_	P_Het_ †	OR (95% CI)	P_Eff_	P_Het_ †
Overall	8		0.87 (0.68–1.12)	0.28	<0.01	0.85 (0.38–1.90)	0.69	<0.01	0.72 (0.49–1.06)	0.10	<0.01	0.66 (0.38–1.16)	0.24	0.05
In HWE*	5		**0.71 (0.60**–**0.83)**	**<0.001**	0.66	**0.58 (0.40**–**0.83)**	**0.003**	0.51	**0.70 (0.57**–**0.85)**	**<0.001**	0.58	**0.45 (0.30**–**0.68)**	**<0.001**	0.80
Subgroup by ethnicity														
EA	3		**0.74 (0.57**–**0.96)**	**0.02**	0.49	**0.32 (0.14**–**0.71)**	**0.005**	0.71	0.79 (0.59–1.06)	0.12	0.31	**0.35 (0.15**–**0.80)**	**0.01**	0.83
WA	3		1.13 (0.57–2.25)	0.73	0.73	2.08 (0.40–10.7)	0.38	<0.01	1.53 (0.20–11.4)	0.68	<0.01	0.97 (0.15–6.25)	0.97	0.01
Subgroup by age														
Adult	2	**0.77 (0.62**–**0.96)**		**0.02**	0.67	1.42 (0.36–5.64)	0.62	<0.01	**0.62 (0.47**–**0.83)**	**0.001**	0.13	0.91 (0.39–2.11)	0.82	0.13
Child	4	1.07 (0.64–1.76)		0.80	<0.01	0.88 (0.18–4.30)	0.87	<0.01	1.09 (0.37–3.20)	0.87	<0.01	0.78 (0.22–2.75)	0.70	0.04
Mixed	2	**0.67 (0.49**–**0.92)**		**0.01**	0.59	**0.28 (0.12**–**0.69)**	**0.006**	0.52	0.72 (0.50–1.04)	0.08	0.21	**0.31 (0.12**–**0.79)**	**0.01**	0.67

*No* number, *Vs.* versus, *OR* odds ratio, *CI* confidential interval, *P_Eff_* P value of pooled effect, *P_Het_* P value of heterogeneity test, *HWE* Hardy-Weinberg equilibrium, *EA* East Asian, *WA* West Asian.

† Random effects model was used when *P_Het_* <0.1; otherwise, fixed effects model was used.

* Three studies of control groups were deviated from Hardy-Weinberg equilibrium.

### IL-10 -819 C/T Polymorphism and Asthma

A summary of findings concerning the associations between IL-10 -819 C/T polymorphism and asthma is shown in Table 3. Four studies with 740 asthmatic subjects and 996 control subjects were included in this meta-analysis. No association was found between asthma and IL-10 -819 C/T polymorphism using the allele contrast, recessive, dominant, or homozygote contrast (OR for C allele = 0.78, 95% CI = 0.58–1.04, p = 0.09) (Table 3). In addition, meta-analysis stratified by age revealed no association between asthma and IL-10 -819 C/T polymorphism (Table 3).

**Table 3:** Meta-analysis of association between the IL-10 -819 C/T polymorphism and asthma.

Population	No	−819 C/T C vs. T			CC vs. CT+TT (Recessive)			CC+CT vs. TT (Dominant)				CC vs. TT (Additive)		
		OR (95% CI)	P_Eff_	P_Het_ †	OR (95% CI)	P_Eff_	P_Het_ †	OR (95% CI)	P_Eff_	P_Het_ †	OR (95% CI)		P_Eff_	P_Het_ †
Overall*	4	0.78 (0.58–1.04)	0.09	0.02	0.54 (0.25–1.16)	0.11	<0.01	0.95 (0.75–1.22)	0.70	0.34	0.84 (0.60–1.18)		0.31	0.68
Subgroup by age														
Adult	2	0.84 (0.69–1.03)	0.09	0.95	0.76 (0.57–1.02)	0.06	0.62	0.84 (0.57–1.23)	0.37	0.71	0.71 (0.46–1.10)		0.13	0.73
Child	2	0.68 (0.30–1.53)	0.35	<0.01	0.20 (0.00–8.61)	0.41	<0.01	1.04 (0.76–1.43)	0.81	0.10	1.07 (0.63–1.84)		0.80	0.90

*No* number, *Vs.* versus, *OR* odds ratio, *CI* confidential interval, *P_Eff_* P value of pooled effect, *P_Het_* P value of heterogeneity test.

† Random effects model was used when *P_Het_* <0.1; otherwise, fixed effects model was used.

* All studies were in Hardy-Weinberg equilibrium in control group.

### IL-10 -592 C/A Polymorphism and Asthma

Six publications (1372 asthmatic subjects and 1266 control subjects) reported on the genotype frequency of IL-10 -592 C/A polymorphism and asthma (Table 4). Meta-analysis of the IL-10 -592 C/A polymorphism showed no association between asthma and the IL-10 -592 C allele in all study subjects (OR = 1.01, 95% CI = 0.66–1.54, p = 0.98) (Table 4). In addition, analysis using the recessive, dominant model, and homozygote contrast showed the same pattern for the IL-10 -592 C allele. Stratification by age indicated no association between IL-10 -592 C/A polymorphism and asthma in adult, children, and mixed groups (Table 4). In the stratified analysis by ethnicity, no association was found between asthma and IL-10 -592 C/A polymorphisms in East Asian, and West Asian groups, only except for West Asians using homozygote contrast (OR = 5.70, 95% CI = 2.38–13.6, p<0.0001) (Table 4).

**Table 4:** Meta-analysis of association between the IL-10 -592 C/A polymorphism and asthma.

Population	No	−592 C/A C vs. A			CC vs. CA+AA (Recessive)			CC+CA vs. AA (Dominant)				CC vs. AA (Additive)		
		OR (95% CI)	P_Eff_	P_Het_ †	OR (95% CI)	P_Eff_	P_Het_ †	OR (95% CI)	P_Eff_	P_Het_ †	OR (95% CI)		P_Eff_	P_Het_ †
Overall*	6	1.01 (0.66–1.54)	0.98	<0.01	0.91 (0.48–1.72)	0.77	<0.01	1.01 (0.89–1.14)	0.92	0.70	1.19 (0.62–2.28)		0.59	<0.01
Subgroup by ethnicity														
EA	2	0.96 (0.80–1.14)	0.64	0.36	0.95 (0.67–1.35)	0.77	0.50	0.97 (0.80–1.17)	0.74	0.55	0.93 (0.63–1.36)		0.69	0.39
WA	2	1.28 (0.14–11.27)	0.83	<0.01	0.35 (0.0–206.1)	0.75	<0.01	1.24 (0.92–1.66)	0.15	0.80	**5.70 (2.38**–**13.6)**		**<0.001**	0.13
Subgroup by age														
Adult	2	0.82 (0.67–1.00)	0.05	0.46	0.79 (0.62–1.02)	0.07	0.50	0.96 (0.78–1.16)	0.65	0.65	0.71 (0.46–1.08)		0.11	0.35
Child	2	0.67 (0.28–1.63)	0.38	<0.01	0.18 (0.0–15.2)	0.45	<0.01	1.06 (0.85–1.33)	0.58	0.56	1.06 (0.63–1.78)		0.82	0.67
Mixed	2	1.83 (0.43–7.82)	0.42	<0.01	1.88 (0.38–9.34)	0.44	<0.01	1.02 (0.81–1.27)	0.89	0.17	2.24 (0.26–19.2)		0.46	<0.01

*No* number, *Vs.* versus, *OR* odds ratio, *CI* confidential interval, *P_Eff_* P value of pooled effect, *P_Het_* P value of heterogeneity test, *EA* East Asian, *WA* West Asian.

† Random effects model was used when *P_Het_* <0.1; otherwise, fixed effects model was used.

* All studies were in Hardy-Weinberg equilibrium in control group.

### IL-10 Promoter Haplotype and Asthma

Three single nucleotide polymorphisms (SNPs) at the promoter region (−1082 G/A, −819 C/T and −592 C/A) were in complete linkage disequilibrium and three haplotypes were present (GCC. ACC. ATA). Seven case-control studies (1802 asthmatic subjects and 1709 control subjects) were included on the relationship between IL-10 promoter haplotype and the susceptibility of asthma (Table 5). Meta-analysis of homozygous GCC, ACC, ATA haplotypes failed to show a significant association with asthma (OR = 0.97, 95% CI = 0.77–1.23, p = 0.81; OR = 1.00, 95% CI = 0.80–1.25, p = 0.97; OR = 1.25, 95% CI = 0.97–1.60, p = 0.08, respectively). Subgroup analysis based on ethnicity and age did not show statistical significance between GCC, ACC, ATA haplotypes and asthma risk (Table 5).

**Table 5:** Meta-analysis of association between the IL-10 promoter haplotype (−1082 G/A, −819 C/T, −592 C/A) and asthma.

Population	No	GCC vs. others			No	ACC vs. others			No	ATA vs. others		
		OR (95% CI)	P_Eff_	P_Het_ †		OR (95% CI)	P_Eff_	P_Het_ †		OR (95% CI)	P_Eff_	P_Het_ †
Overall*	6	0.97 (0.77–1.23)	0.81	<0.01	7	1.00 (0.80–1.25)	0.97	<0.01	6	1.25 (0.97–1.60)	0.08	<0.01
Subgroup by ethnicity												
EU	3	0.86 (0.65–1.14)	0.28	0.05	3	1.03 (0.87–1.22)	0.70	0.78	3	1.14 (0.95–1.36)	0.17	0.11
EA	1	NA	NA	NA	2	1.08 (0.89–1.31)	0.43	0.47	1	NA	NA	NA
Subgroup by age												
Adult	3	0.95 (0.81–1.11)	0.51	0.30	3	1.00 (0.86–1.17)	0.97	0.84	3	1.16 (0.99–1.37)	0.06	0.10
Child	2	1.37 (0.74–2.52)	0.32	0.05	2	0.15 (0.0–22.1)	0.45	<0.01	2	1.41 (0.54–3.65)	0.48	<0.01
Mixed	1	NA	NA	NA	2	1.04 (0.85–1.27)	0.71	0.70	1	NA	NA	NA

*No* number, *Vs.* versus, *OR* odds ratio, *CI* confidential interval, *P_Eff_* P value of pooled effect, *P_Het_* P value of heterogeneity test, *EU* European, *EA* East Asian, *NA* not applicable.

† Random effects model was used when *P_Het_* <0.1; otherwise, fixed effects model was used.

* One study was only available for ACC haplotype frequency [21].

### Heterogeneity and Publication Bias

Some between-study heterogeneity was found during the meta-analyses, but no evidence of heterogeneity was found for analyses of IL-10 -1082 G/A, -819 C/T and -592 C/A polymorphisms. It was difficult to correlate the funnel plot, which is usually used to detect publication bias, as the number of studies included in the analysis was small. Eggers’s regression test showed no evidence of publication bias in this meta-analysis (Egger’s regression test p>0.1). The distributions of genotypes in the normal control groups were not consistent with the H-W equilibrium in three studies of the IL-10 -1082 G/A polymorphism [17]–[19]. Deviation from H-W equilibrium among controls implies potential bias during control selection, or genotyping errors, but excluding these studies did affect our result for an association between the IL-10 -1082 G/A polymorphism and asthma (Table 2, Figure 2). All studies were in H-W equilibrium for the IL-10 -819 C/T and -592 C/A polymorphisms.

## Discussion

IL-10 is a potent immunomodulatory molecule that inhibits the synthesis of pro-inflammatory cytokines, and upregulates of B cell production and differentiation [34]. Its association of airway tone regulation is evident from various observations including human and animal model [35], [36]. Low levels of IL-10 expression have a role on the pathogenesis of asthma [37], [38]. On the other hand, high level of IL-10 from regulatory T cells has a protective effect against airway hyperreactivity and inflammation [39]. Since cytokine production is genetically controlled at the transcription level, the association of IL-10 polymorphisms at the genotypic or haplotypic levels with asthma has been reported in a numerous studies [14]–[24]. Three SNPs (-1082 G/A, -819 C/T, and -592 C/A) have been implicated as potential risk factors for asthma [7], [8].

In the present study, we addressed associations between the IL-10 -1082 G/A, -819 C/T, -592 C/A polymorphisms, and their haplotypes and asthma susceptibility. The IL-10 -1082 G/A polymorphism conferred susceptibility to asthma in East Asians and adult asthmatics. However, the meta-analysis revealed no association between IL-10 -819 C/T, -592 C/A polymorphisms, and their haplotypes and asthma.

In the polymorphism at position -1082 G/A, which lies within a putative ETS-like transcription factor binding site, it has been reported that G position may be associated with a higher expression of the IL-10 gene [40], [41]. Clinically, a suggestive association between -1082 G/A and both IgE, FEV_1_ as a percentage of predicted (FEV_1_PP) has been reported [12]. The data demonstrated in this meta-analysis should be interpreted with some caution. Even though we revealed significant associations by sensitivity analysis for studies in H-W equilibrium, which excluded potential bias during the control selection, there were only five studies were in H-W equilibrium, which is a limited population. Discrepancy remained between East or West Asians, as well as adult or child population. The relative importance of the -1082 G/A polymorphisms during the development of asthma may vary between ethnic and age groups, but we did not perform subgroup meta-analysis on the -1082 G/A polymorphism in H-W equilibrium due to limited data.

Besides, we failed to find any association between IL-10 -819 C/T and -592 C/A polymorphisms and asthma risk under different contrast models with subgroup analysis on the basis of age and ethnicity. In addition, haplotype analysis which has provided more information than single polymorphism showed that no evidence of association was found between the haplotypes of the GCC, ACC, ATA and asthma. A previous study concerning the function of SNPs of regulatory T cells showed that analyses of gene-to-gene interactions about –819T/C and –592A/C polymorphisms did not show any association with asthma and atopy that supporting this result [42]. It seems -819 C/T and -592 C/A were relatively less affect to asthma unlike other inflammatory disease including rheumatoid arthritis and systemic sclerosis [43], [44]. It has been shown that the effect of the ATA haplotype on asthma in Caucasian and Finnish populations with various eosinophilic count and serum IgE levels is contentious [22], [24]. The conclusion that -819 C/T and 592 C/A polymorphisms and ATA haplotype were of no role in asthma risk could be premature due to limited number of studies included in this meta-analysis. There should be additional large scale studies about asthma to clarify these relationships.

There are several limitations that should be considered when interpreting our results. Heterogeneity and confounding factors may have affected the meta-analysis. Because asthma itself is heterogeneous disease, asthma phenotype should be fully specified. Divergent patient group inclusion criteria and enrollment of control group without asthma screening test could have distorted the results. Publication bias may also have affected the analysis. Since studies that produced negative results may not have been published, we could not eliminate the possibility of bias even though we performed Egger’s regression test. Finally, IL-10 polymorphisms may be associated with asthma severity as well as susceptibility. However, the small amount of data available did not allow us to perform meta-analysis this association.

In conclusion, this meta-analysis demonstrates that IL-10 -1082 G/A polymorphism confers susceptibility to asthma in East Asians and adult asthmatics. However, IL-10 -819 C/T, -592 C/A polymorphisms, and their haplotypes were not associated with asthma. Further well designed high quality researches with larger scale and various ethnicities are required to explore the potential roles of these polymorphisms and pathogeneses of asthma.

## Supporting Information

Supplement S1 **Search strategy for meta-analysis of association between interleukin-10 gene polymorphisms and the risk of asthma.**
(DOCX)

Supplement S2 **PRISMA 2009 Checklist of items to include when reporting a systematic review or meta-analysis.**
(DOC)

Supplement S3 **The flow chart of the included studies in accordance to the PRISMA 2009 statement.**
(DOC)

Supplement S4 **Criteria of methodological quality assessment for molecular association case/control study for asthma.**
(DOCX)

Supplement S5 **Quality score assessment results.**
(DOCX)
