# Supplementary material for: Interleukin-10 Promoter Gene Polymorphisms and Susceptibility to Asthma: A Meta-Analysis
Source: PLoS One. 2013 Jan 15;8(1):e53758. doi: 10.1371/journal.pone.0053758 (PMC3546046; doi:10.1371/journal.pone.0053758)
Supplement: Supplement S1 — Search strategy for meta-analysis of association between interleukin-10 gene polymorphisms and the risk of asthma. (DOCX) [file pone.0053758.s001.docx]

**Supplement S1.** Search strategy for meta-analysis of association between interleukin-10 gene polymorphisms and the risk of asthma

**MEDLINE**

1. “interleukin-10”[MH]
2. “interleukin 10”[ALL] OR “IL-10”[ALL] OR “IL10”[ALL]
3. 1 or 2
4. “polymorphism, genetic”[MH] OR “polymorphism, single nucleotide”[MH]
5. “polymorphism, genetic”[ALL] OR “polymorphism, single nucleotide”[ALL] OR “SNP”[ALL] OR “polymorphism*”[ALL] OR “variant*”[ALL] OR “mutation*”[ALL] OR “mutant*”[ALL]
6. 4 OR 5
7. 3 AND 6
8. “asthma”[MH]
9. “asthma*”[ALL] OR “bronchial asthma”[ALL] OR “bronchospas*”[ALL] OR “bronchial spasm”[ALL] OR “bronchoconstrict*”[ALL] OR “bronchial hyperreactivity”[ALL] OR “respiratory hypersensitivity”[ALL] OR “wheez*”[ALL]
10. 8 OR 9
11. 7 AND 10
12. “humans”[MH]
13. 11 AND 12

MH = MeSH terms, ALL = All fields

**Limitation:** humans

**Date of Search:** Aug. 31 2012

**Results:** 61 articles were found

**EMBASE**

1. ‘interleukin-10’/exp OR ‘interleukin-10’/syn
2. ‘IL-10’ OR ‘IL10’
3. 1 OR 2
4. ‘genetic polymorphism’/exp OR ‘genetic polymorphism’/syn
5. ‘single nucleotide polymorphism’/exp OR ‘single nucleotide polymorphism’/syn
6. ‘DNA polymorphism’/syn OR ‘polymophisms’ OR ‘polymophism’ OR ‘SNPs’ OR ‘SNP’ OR ‘variants’ OR ‘variant’ OR ‘mutation’/syn OR ‘mutant’/syn
7. 4 OR 5 OR 6
8. 3 AND 7
9. ‘asthma’/exp OR ‘asthma’/syn
10. ‘bronchial asthma’/syn OR ‘bronchospasm’/syn OR ‘bronchus hyperreactivity’/syn OR ‘bronchoconstriction’/syn OR ‘allergic asthma’/syn OR ‘respiratory tract allergy’/syn OR ‘wheezing’/syn
11. 9 OR 10
12. 8 and 11
13. [humans]/lim
14. 12 and 13

Exp = explosion search, syn = synonymous search, lim = limitation

**Limitation:** humans

**Date of Search:** Aug. 31 2012

**Results:** 249 articles were found

**CENTRAL database (The Cochrane Library)**

1. MeSH descriptor **Interleukin-10** explode all trees
2. (IL10) or (IL-10)
3. #1 OR #2
4. Mesh descriptor **Polymorphism, Single Nucleotide** explode all tress
5. Mesh descriptor **Polymorphism, Genetic** explode all tress
6. (genetic polymorphism) OR (single nucleotide polymorphism) OR (polymorphism*) OR (SNP*) OR (variant*) OR (mutation*) OR (mutant*)
7. #4 OR #5 OR #6
8. #3 AND #7
9. Mesh descriptor **Asthma** explode all tress
10. (asthma*) OR (bronchial asthma*) OR (bronchial spas*) OR (bronchial hyperreactiv*) OR (bronchospasm*) OR (bronchoconstrict*) OR (allergic asthma) OR (respiratory tract allergy) OR (wheez*)
11. #9 OR #10
12. #8 AND # 11

All field text searched

**Limitation:** none

**Date of Search:** Aug. 31 2012

**Results:** 10 articles were found

A total 320 articles were screened
