# Supplementary material for: Interleukin-10 Promoter Gene Polymorphisms and Susceptibility to Asthma: A Meta-Analysis
Source: PLoS One. 2013 Jan 15;8(1):e53758. doi: 10.1371/journal.pone.0053758 (PMC3546046; doi:10.1371/journal.pone.0053758)
Supplement: Supplement S3 — The flow chart of the included studies in accordance to the PRISMA 2009 statement. (DOC) [file pone.0053758.s003.doc]

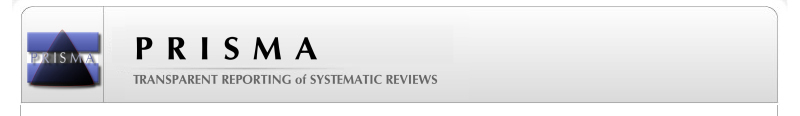
**PRISMA 2009 Flow Diagram**

**Screening**

**Included**

**Eligibility**

**Identification**

Records identified through database searching
(n = 320)

Additional records identified through other sources
(n = 2)

Records after duplicates removed
(n = 265)

Records screened
(n = 265)

Records excluded for irrelevance (n = 242)

Full-text articles assessed for eligibility
(n = 23)

Records excluded for no detailed genotype, no case control study, and familial base study
(n = 12)

Studies included in qualitative synthesis
(n = 11)

Records excluded for repeated publication
(n = 0)
