# Supplementary material for: Interleukin-10 Promoter Gene Polymorphisms and Susceptibility to Asthma: A Meta-Analysis
Source: PLoS One. 2013 Jan 15;8(1):e53758. doi: 10.1371/journal.pone.0053758 (PMC3546046; doi:10.1371/journal.pone.0053758)
Supplement: Supplement S4 — Criteria of methodological quality assessment for molecular association case/control study for asthma. (DOCX) [file pone.0053758.s004.docx]

**Supplement S4.** Criteria of methodological quality assessment for molecular association case/control study for asthma

| Criteria |  | Score |
| --- | --- | --- |
| A | Representativeness of cases |  |
|  | Consecutive/randomly selected from case population with clearly defined sampling frame | 2 |
|  | Consecutive/randomly selected from case population without clearly defined frame or extensive inclusion/exclusion criteria | 1 |
|  | No method of selection described | 0 |
| B | Representativeness of controls |  |
|  | Controls were consecutive/randomly drawn from the same sampling frame (ward/community) as cases | 2 |
|  | Controls were consecutive/randomly drawn from a different sampling frame as cases | 1 |
|  | Not described | 0 |
| C | Ascertainment of asthma |  |
|  | Clearly described objective criteria for diagnosis of asthma | 2 |
|  | Diagnosis of asthma by patient self-report or by patient history | 1 |
|  | Not described | 0 |
| D | Ascertainment of controls |  |
|  | Controls were tested to screen out asthma, i.e., measured FEV1 or PEFR | 2 |
|  | Controls were subjects who did not report asthma; no objecting test | 1 |
|  | Not described | 0 |
| E | Genotyping examination |  |
|  | Genotyping done under “blinded” condition | 1 |
|  | Unblinded or not mentioned | 0 |
| F | HWE |  |
|  | HWE in control group | 2 |
|  | HWE in deviation in control group | 1 |
| G | Association assessment |  |
|  | Assess association between genotypes and asthma with appropriate statistics and adjustment for confounders | 2 |
|  | Assess association between genotypes and asthma with appropriate statistics and without adjustment for confounders | 1 |
|  | Inappropriate statistics used | 0 |

*FEV1* forced expiratory volume in 1 second, *HWE* Hardy-Weinberg equilibrium, *PEFR* peak expiratory flow rate

Quality assessment scoring system according to Thakkinstian et al [[1](#_ENREF_1)].

1. Thakkinstian A, McEvoy M, Minelli C, Gibson P, Hancox B, et al. (2005) Systematic review and meta-analysis of the association between {beta}2-adrenoceptor polymorphisms and asthma: a HuGE review. Am J Epidemiol 162: 201-211.
