# Supplementary material for: Interleukin-10 Promoter Gene Polymorphisms and Susceptibility to Asthma: A Meta-Analysis
Source: PLoS One. 2013 Jan 15;8(1):e53758. doi: 10.1371/journal.pone.0053758 (PMC3546046; doi:10.1371/journal.pone.0053758)
Supplement: Supplement S5 — Quality score assessment results. (DOCX) [file pone.0053758.s005.docx]

**Supplement S5.** Quality score assessment results

| Study | A | B | C | D | E | F | G | Total |
| --- | --- | --- | --- | --- | --- | --- | --- | --- |
| Hakimizadeh et al. 2012 | 2 | 2 | 2 | 2 | 0 | 2 | 1 | 11 |
| Hussein et al. 2011 | 0 | 1 | 2 | 2 | 0 | 2 | 1 | 8 |
| Kim et al. 2011 | 0 | 1 | 2 | 2 | 0 | 2 | 2 | 9 |
| Trajkov et al. 2009 | 2 | 1 | 2 | 0 | 0 | 1 | 1 | 7 |
| Movahedi et al. 2008 | 2 | 1 | 2 | 0 | 0 | 1 | 1 | 7 |
| Zedan et al. 2008 | 2 | 2 | 2 | 1 | 0 | 1 | 1 | 9 |
| Chatterjee et al. 2005 | 2 | 2 | 2 | 2 | 0 | 2 | 1 | 11 |
| Park et al. 2004 | 2 | 2 | 2 | 2 | 0 | 2 | 2 | 12 |
| Karjalainen et al. 2003 | 2 | 2 | 2 | 2 | 0 | NA | 1 | 9 |
| Hang et al. 2003 | 0 | 0 | 2 | 2 | 0 | 2 | 1 | 7 |
| Lim et al. 1998 | 0 | 2 | 2 | 1 | 0 | NA | 1 | 6 |

*NA* not applicable

Quality assessment scoring system according to Thakkinstian et al [[1](#_ENREF_1)].

1. Thakkinstian A, McEvoy M, Minelli C, Gibson P, Hancox B, et al. (2005) Systematic review and meta-analysis of the association between {beta}2-adrenoceptor polymorphisms and asthma: a HuGE review. Am J Epidemiol 162: 201-211.
